# Subclinical postoperative atrial fibrillation: a randomized trial

**DOI:** 10.3389/fcvm.2023.1153275

**Published:** 2023-05-25

**Authors:** Avi Sabbag, Anat Berkovich, Ehud Raanani, David Volvovitch, William F. McIntyre, Yigal Kassif, Alexander Kogan, Michael Glikson, Roy Beinart

**Affiliations:** ^1^ Davidai Arrhythmia Center, Sheba Medical Center, Ramat Gan, Israel; ^2^ Sackler Faculty of Medicine, Tel Aviv University, Tel Aviv, Israel; ^3^ Department of Cardiac Surgery, Sheba Medical Center, Ramat Gan, Israel; ^4^ Population Health Research Institute, McMaster University, Hamilton, ON, Canada; ^5^ Jesselson Integrated Heart Center, Shaare Zedek Medical Center, Jerusalem, Israel

**Keywords:** post-operative atrial fibrillation, implantable loop recorder, stroke, atrial fibrillation, cardiac surgery

## Abstract

**Background:**

Postoperative atrial fibrillation (POAF) is the most common complication of cardiac surgery, requiring interventions and prolonging hospital stay. POAF is associated with increased mortality and a higher rate of systemic thrombo-embolism. The rates of recurrent AF, optimal follow-up and management remain unclear. We aimed to evaluate the incidence of recurrent atrial fibrillation (AF) events, during long term follow-up in patients with POAF following cardiac surgery.

**Methods:**

Patients with POAF and a CHA_2_DS_2_-VASc score of ≥2 were randomized in a 2:1 ratio to either implantation of a loop recorder (ILR) or ECG monitoring using periodic Holters. Participants were followed prospectively for 2 years. The primary end point was the occurrence of AF longer than 5 min.

**Results:**

The final cohort comprised of 22 patients, of whom 14 received an ILR. Over a median follow up of 25.7 (IQR of 24.7–44.4) months, 8 patients developed AF, representing a cumulative annualized risk of AF recurrence of 35.7%. There was no difference between ILR (6 participants, 40%) and ECG/Holter (2 participants, 25% *p* = 0.917). All 8 patients with AF recurrence were treated with oral anticoagulation. There were no cases of mortality, stroke or major bleeding. Two patients underwent ILR explantation due to pain at the implantation site.

**Conclusions:**

The rate of recurrent AF in patients with POAF after cardiac surgery and a CHA_2_DS_2_-VASc score of ≥2 is approximately 1 in 3 when followed systematically. Further research is need to assess the role of ILRs in this population.

## Introduction

Postoperative atrial fibrillation (POAF), defined as new-onset atrial fibrillation (AF) in the immediate period after surgery (1, 2), is the most common complication of cardiac surgery (3, 4). Affecting 10%–65% of patients (3, 5), this arrhythmia is associated with increased mortality, and morbidity including stroke and hemodynamic deterioration (6). POAF prolongs the hospital stay and increases health costs. The incidence of POAF is increasing (7), resulting from an increase in the age and burden of arrhythmic risk factors (3) in patients undergoing cardiac surgery.

Despite the high incidence of POAF, its association with recurrent AF after hospital discharge remains unclear. There is uncertainty with respect to the long term follow up and management of these patients.

The first aim of the current study was to assess the incidence of recurrent AF events, during long term follow-up in patients that presented with POAF following cardiac surgery and were discharged in sinus rhythm. Secondly, we aimed to assess the efficacy of an implanted cardiac monitor, as compared to usual care, to detect recurrent AF events during follow-up.

## Methods

The study was conducted at 2 tertiary medical centers in Israel (Sheba medical center and Shaare Zedek Medical Center) from August 2017 through March 2021 (NCT 02522364). We recruited adult patients with documented new-onset AF lasting at least 5 min occurring during the index hospitalization following cardiac surgery. Furthermore, patients were required to have a CHA_2_DS_2_-VASc score of 2 or higher. Consenting participants were randomized in a 1:2 ratio to either ECG monitoring using periodic Holters (at 2 and 6 months post discharge) alone (No-ILR) and periodic Holters plus implantation of a loop recorder (ILR). The main exclusion criteria were a contraindication for oral anticoagulation, a dual chamber cardiac implantable device and active systemic infection.

All participants were discharged in sinus rhythm and were followed prospectively for a minimum of 2 years. The follow up included Holter monitoring at 3 and 6 months post discharge and clinic visits biannually. Patients randomized to ILR were implanted with a Biomonitor 2 device (Biotronik, Berlin, Germany) within 2 weeks of surgery. Participants were also connected to a remote monitoring system, allowing for continuous surveillance of arrhythmic events. All patients were treated with apixaban for a minimum of 3 months. Treatment with anticoagulation was at the discretion of the attending physician but would be continued to a maximum duration of 6 weeks after hospital discharge unless AF reoccurred. Any documentation of recurrent AF of ≥5 min would trigger continuation or re-initiation of OAC.

The primary end point was the occurrence of AF lasting at least 5 min. Additional endpoints were all-cause death, stroke, rapid AF requiring hospitalization and initiation of long term anticoagulation. The main safety end points were acute complication of ILR implantation and major bleeding.

Results are presented as median [interquartile-range (IRQ)] or mean ± SD as appropriate. Continues variables were compared using a student-*t* test or Wilcoxon test and binary variables were compared using *χ*^2^ tests. Predictors of AF recurrence were evaluated by a univariable cox proportional hazards model followed by a multivariable model. Statistical analysis was carried out using SPSS v21 (Chicago, Ill., USA). A two-sided *p* value <0.05 was considered statistically significant.

The study was approved by local institutional ethical boards and complied with the Declaration of Helsinki. All study participants gave written informed consent.

## Figures and Tables

**Figure 1:**
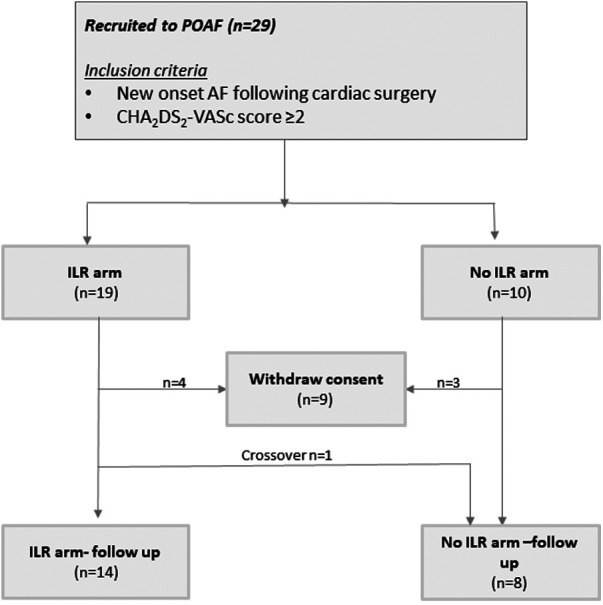
Patient flow. AF, atrial fibrillation; ILR, implantable loop recorder; POAF, post operative atrial fibrillation.

**Table 1:** Baseline characteristics—as treated (participants that continued with follow up).

	All (*n* = 22)	ILR (*n* = 14)	No-ILR (8)
Age	66 (64–73)	66.5 (64–73)	66 (64–73)
Male	17 (77)	11 (78.6)	6 (75)
Ischemic heart disease	15 (68.2)	10 (71.4)	5 (62.5)
Hypertension	19 (86.4)	12 (85.7)	7 (87.5)
Diabetes mellitus, insulin dependent	1 (4.5)	1 (7.1)	0
Diabetes mellitus, non-insulin dependent	7 (31.8)	3 (21.4)	4 (50)
Chronic obstructive pulmonary disease	4 (18.2)	3 (21.4)	1 (12.5)
Stroke	3 (13.6)	1 (7.1)	2 (25)
Transient ischemic accident	1 (4.5)	1 (7.1)	0
Malignancy	2 (9.1)	1 (7.1)	1 (12.5)
Heart failure			
Chronic kidney disease	2 (9.1)	1 (7.1)	1 (12.5)
Active smoking	3 (13.6)	2 (14.3)	1 (12.5)
Chronic medical therapy			
Aspirin	16 (72.7)	11 (78.6)	5 (62.5)
Clopidogrel	6 (27.3)	4 (28.6)	2 (25)
Any Antipletelet			
Beta blocker	11 (50)	6 (42.9)	5 (62.5)
ACE inhibitor/ARB	13 (59.1)	9 (64.3)	4 (50)
PR duration	158 (136–193)	166 (137–199)	156 (136–181)
QRS duration	90 (85–99)	88 (79–96)	95 (91–111)
QTc	425 (413–449)	420 (411–442)	438 (416–476)
Baseline echo			
LVEF, median (IQR), %	60 (55–60)	60 (55–60)	60 (57–65)
LA diameter, median (IQR), mm	42 (36–46)	40 (34–46)	43 (39–47)
SPAP			
AS ≥ Moderate	5 (33.3)	3 (33.3)	2 (33.3)
MR ≥ Moderate	4 (26.7)	2 (20)	2 (40)
Lab			
Hemoglobin (g/dl)	12.3 (11–14)	12.1 (10.8–14.1)	12.7 (11.6–14)
Creatinine mg/dl	0.87 (0.67–1.06)	0.92 (0.8–1.06)	0.69 (0.6–1.21)
Surgery type			
CABG	15 (68.2)	9 (64.3)	6 (75)
AVR	5 (22.7)	4 (28.6)	1 (12.5)
Mitral valve repair	2 (9.1)	1 (7.1)	1 (12.5)
MVR to bio-prosthesis	2 (9.1)	1 (7.1)	1 (12.5)
Urgent surgery	9 (45)	3 (25)	6 (75)
Total hospitalization duration	10 (7.7–15.2)	11 (7.7–16)	10 (7–14)
Electrical cardioversion	3 (14.3)	2 (12.5)	1 (13.6)
Amiodarone at discharge	15 (71.4)	8 (61.5)	7 (87.5)

ACE, angiotensin-converting-enzyme; ARB, angiotensin receptor blockers; AVR, aortic valve replacement; AS, aortic stenosis; CABG, coronary artery bypass surgery; ILR, implantable loop recorder; LA, left atrium; LVEF, left ventricular ejection fraction; MR, mitral regurgitation; MVR, mitral valve replacement; SPAP, systolic pulmonary artery pressure.

**Table 2:** Time to first diagnosis of atrial fibrillation.

Group	Surgery	Urgency level	Time to first AF (days)	Re-initiation of OAC
ILR	CABG	Urgent	8	Never stopped
ILR	AVR	Elective	115	118
ILR	MV repair	Elective	117	121
ILR	CABG	Elective	191	194
ILR	CABG	Elective	549	555
ILR	AVR	Urgent	14	Never stopped
No ILR	CABG + AVR	Urgent	83	90
No ILR	MVR	Elective	78	89

AVR, aortic valve replacement; ILR, implantable loop recorder; MV, mitral valve; OAC, oral anticoagulation.

**Table 3:** Predictors of atrial fibrillation recurren.

Univariable predictors of AF recurrence		
Variable	Hazard rations	*p* value
Age	1.03 (0.94–1.13)	0.502
Sex	1.02 (0.2–5.08)	0.981
PR duration (per ms)	1.01 (0.99–1.03)	0.372
QRS duration (per ms)	1.00 (0.95–1.05)	0.911
LVEF, % (per %)	0.97 (0.79–1.17)	0.731
Urgent surgery	1.86 (0.36–9.61)	0.455
MV surgery	**24.73 (2.18–280)**	**0.01**
AV surgery	0.95 (0.43–2.12)	0.905
CABG surgery	0.31 (0.7–1.4)	0.128
MR severity (per level)	**3.5 (1.21–10)**	**0.021**
ILR	1.4 (0.28–7.1)	0.680
Age and sex-adjusted analysis		
MV surgery	**30 (2.17–414)**	**0.011**

Statistically significant compressions are presented in bold.

AV, aortic valve; CABG, coronary artery bypass grafting; LVEF, left ventricular ejection fraction; MV, mitral valve.

## Data Availability

The raw data supporting the conclusions of this article will be made available by the authors, without undue reservation.

## Results

A total of 29 patients were recruited (see Supplementary Table for baseline characteristics), among whom 7 withdrew consent after randomization. One participant that was randomized to ILR crossed over to the no-ILR arm (Figure 1). The final study cohort was comprised of 22 participants {median age 66 [interquartile range (IQR) 64–73], 23% female}, of whom 14 underwent ILR implantation.

All participants in the study were treated with amiodarone during their hospitalization. This resulted in cardioversion in 20 (90) % the reminder underwent electrical cardioversion. All patients were discharged in sinus rhythm (as required by the study protocol) and 7 were discharged with amiodarone. The baseline characteristics of the as-treated cohort are detailed in Table 1.

Over a median follow up of 25.7 (IQR 24.7–44.4) months, 8 patients developed AF, representing a cumulative risk of 33.8% (23.3%–44.4%) of AF recurrence at 1 year. Six (40%) were diagnosed by ILR recording while 2 (25%) by ECGs or Holter monitoring (Table 2). The median time to first AF detection was 99 (IQR 30, 172) days and was similar in both groups (log rank *p* = 0.690). All 8 participants were treated with oral anticoagulation (OAC). The patients that had early AF recurrences continued OAC throughout the study. In the remaining 6, the treatment was re-initiated within a median of 5 (3–11) days from detection of AF recurrence. Treatment with amiodarone was stopped after 6 weeks in all but 2 patients who had early AF recurrence.

In our cohort, the risk of AF recurrence was not affected by sex, age nor the urgency of the surgery (Table 3). The risk was higher in patients that underwent mitral valve surgery (mitral valve repair or replacement to a biological valve). The association remained statistically significant after adjustment for age and sex [OR 2.18 (2.18–414)]. Similarly the risk of AF recurrence increased in proportion to the severity of MR [OR 2.18 (2.18–414)].

There were no cases of mortality, stroke or systemic embolism during follow up. Two patients underwent ILR explanation; one due to pain at the implant site and the other as part of treatment of deep sternal infection attributed to the index surgery.

## Discussion

Our study showed a high rate of AF recurrence in patients that developed new onset AF following cardiac surgery. Although the crude rates of AF detection were higher in patients who received an ILR, this difference was not statistically significant. Most AF recurrences were observed within 6 months of the surgery suggesting that intense arrhythmic monitoring during this period may be effective. As defined by the study protocol, all study participants had a CHA_2_DS_2_-VASc score ≥2, therefore each detection of AF recurrence resulted in initiation of OAC therapy.

Our results add to the growing body of evidence that a substantial proportion of patients with POAF develop recurrent AF over time. The recurrence rate in our study (37.5%) is higher than what was observed during long-term follow-up of 3,023 patients in the Arterial Revascularization Trial (18.5% over a median of 6 years) (6). The higher rate observed in our study may be explained by the systematic use of continuous monitoring for recurrent AF.

The long-held notion that POAF is a benign phenomenon is not supported by contemporary data. Multiple studies have shown increased risk of stroke, heart failure or death (6, 8, 9). Yet, the association between POAF and adverse events appears to be stronger early after surgery (9). This, taken with recurrence rates below 50% suggest that identification of POAF alone is not sufficient to identify patients that would benefit from lifelong OAC therapy. This is further supported by a systematic review that failed to show a decrease in the risk of thromboembolic events with the use of OAC following POAF (10) and is reflected by the contemporary European Society of Cardiology Guidelines (1). At this time there are not clear data to guide the risk stratification and selection of patients with POAF that would benefit from OAC. Therefore, systematic follow-up for recurrent AF may be the appropriate strategy. In addition to provision of OAC, such a strategy may identify patients who stand to benefit from rhythm control. The importance of a timely diagnosis of AF is further evident by the results of the recent EAST-AFNET 4 (11) study showing that early implementation of a rhythm control strategy leads to improved outcome, irrespective of AF related symptoms.

Consistent with previous studies (12, 13) the risk of AF recurrence after POAF was high among patients that underwent mitral valve repair or replacement. All of these patients had hemodynamically significant MR that may lead to dilatation and remodeling of the left atrium, creating substrate for AF. Both severe MR and MR surgery may serve as important markers for use in risk stratification.

The results of our study alongside similar other studies emphasize the need for a large prospective randomized study that would be powered not only to more precisely estimate the incidence of AF recurrence and identify its predictors but also to evaluate the potential clinical benefit of early detection of AF recurrence and the impact on clinical outcomes of patients with POAF. Such a study could guide our approach to monitoring these patients, test the incremental value of ILRs over periodic Holters and help select patients that would benefit from life-long OAC. A high-yield population on which to focus may be those undergoing mitral valve surgery.

The main strengths of this study are the randomized design, pre-registration and systematic follow-up. Furthermore, the use of ILR ensures that all AF events were captured, yielding the true incidence of progression from POAF to AF the majority of the study cohort (ILR arm). The study has important limitations. The small sample size, withdrawal of 7 participants, plus the crossover of one study participant to the no-ILR arm may have obscured between group differences. Furthermore, the study is unable to identify specific predictors of AF recurrence.

## Conclusions

Among patients with POAF after cardiac surgery and a CHA_2_DS_2_-VASc score of 2 or more, the rate of AF recurrence as detected with systematic follow up is approximately 1 in 3. In the present study, ILR monitoring did not result in higher rates of AF detection, however between-group differences were likely obscured by small sample sizes.
