# Supplementary material for: Subclinical postoperative atrial fibrillation: a randomized trial
Source: Front Cardiovasc Med. 2023 May 25;10:1153275. doi: 10.3389/fcvm.2023.1153275 (PMC10248069; doi:10.3389/fcvm.2023.1153275)
Supplement: Supplementary file 1 [file Table1.docx]

Supplementary Table - Baseline characteristic – intention to treat (entire cohort)

|  | *All (n=29)* | *ILR (n=19)* | *No-ILR (10)* |
| --- | --- | --- | --- |
| Age, median (IQR) | 68 (64-73) | 68 (64-73) | 68 (64-76) |
| Male (%) | 19 (65.5) | 6 (60) | 4 (40) |
| Ischemic heart disease | 16 (64) | 12 (70.6) | 4 (50) |
| Hypertension | 21 (84) | 14 (87.5) | 7 (87.5) |
| Diabetes mellitus, Insulin dependent | 1 (4) | 1 (5.9) | 0 |
| Diabetes mellitus, non-Insulin dependent | 9 (36) | 4 (23.5) | 5 (62.5) |
| Chronic obstructive pulmonary disease | 4 (16) | 3 (17.6) | 1 (12.5) |
| Stroke | 4 (16) | 2 (11.8) | 2 (25) |
| Transient ischemic accident | 1 (3.8) | 1 (5.6) | 0 |
| Malignancy | 2 (8) | 1 (5.9) | 1 (12.5) |
| Heart failure | 7 (36.8) | 6 (42.8) | 1 (20) |
| Chronic kidney disease | 2 (7.7) | 1 (5.9) | 1 (11.1) |
| Active smoking | 3 (12) | 2 (11.8) | 1 (12.5) |
| Chronic medical Therapy | | | |
| Aspirin | 17 (68) | 13 (76.5) | 4 (50) |
| Clopidogrel | 6 (24) | 5 (29.4) | 1 (12.5) |
| Any Antipletelet |  |  |  |
| Beta blocker | 13 (52) | 8 (47.1) | 5 (62.5) |
| ACE inhibitor/ARB | 15 (60) | 10 (58.8) | 5 (62.5) |
| Baseline ECG | | | |
| PR duration, median (IQR) | 160 (137-195) | 160 (141-199) | 159 (136-183) |
| QRS duration, median (IQR) | 90 (80-99) | 89 (78-96) | 95 (88-111) |
| QTc, median (IQR) | 430 (415-449) | 420 (412-445) | 440 (420-476) |
| Baseline echo |  |  |  |
| LVEF, median (IQR), % | 60 (55-60) | 60 (55-60) | 62 (57-65) |
| LA diameter, median (IQR), mm | 42 (36-46) | 38 (31-45) | 22 (19-30) |
| SPAP , median (IQR) | 38 (28-65) | 30 (24-45) | 41 (36-83) |
| Aortic Stenosis ≥Moderate | 6 (37.5) | 3 (30) | 3 (50) |
| Mitral Regurgitation ≥Moderate | 5 (31.3) | 2 (18.2) | 3 (60) |
| Lab |  |  |  |
| Hemoglobin (g/dL), mean ±SD | 12 ±2 | 12 ±2.2 | 12 ±1.5 |
| Creatinine mg/dL, mean ±SD | 0.9 ±0.4 | 0.9 ±0.4 | 0.8 ±0.3 |
| Surgery type |  |  |  |
| CABG | 16 (55.2) | 11 (57.9) | 5 (50) |
| Aortic valve replacement | 6 (20.7) | 4 (66.7) | 2 (33.3) |
| Mitral valve repair | 2 (6.9) | 1 (5.3) | 1 (10) |
| MVR to bio-prosthesis | 3 (10.3) | 1 (5.3) | 2 (20) |
| Urgent surgery | 11 (47.8) | 5 (33.3) | 6 (75) |
| Total hospitalization duration | 10 (8-15) | 9 (8-17) | 10.5 (7-14) |

Abbreviations: CABG – coronary artery bypass surgery, LA- left atrium, LVEF – left ventricular ejection fraction, MVR – mitral valve replacement
